# Interferon-Gamma Increases the Ratio of Matrix Metalloproteinase-9/Tissue Inhibitor of Metalloproteinase-1 in Peripheral Monocytes from Patients with Coronary Artery Disease

**DOI:** 10.1371/journal.pone.0072291

**Published:** 2013-08-12

**Authors:** Rashidi Springall, Luis M. Amezcua-Guerra, Hector Gonzalez-Pacheco, Janette Furuzawa-Carballeda, Lorena Gomez-Garcia, Ricardo Marquez-Velasco, Ana María Mejía-Domínguez, Jorge Cossío-Aranda, Carlos Martínez-Sánchez, Rafael Bojalil

**Affiliations:** 1 Department of Immunology, Instituto Nacional de Cardiología Ignacio, Chávez, Mexico City, Mexico; 2 Coronary Care Unit, Instituto Nacional de Cardiología Ignacio, Chávez, Mexico City, Mexico; 3 Department of Immunology and Rheumatology, Instituto Nacional de Ciencias Médicas y Nutrición Salvador Zubirán, Mexico City, Mexico; 4 Blood Bank Unit, Instituto Nacional de Cardiología Ignacio, Chávez, Mexico City, Mexico; 5 Cardiology Outpatient Clinic, Instituto Nacional de Cardiología Ignacio, Chávez, Mexico City, Mexico; 6 Department of Health Care, Universidad Autónoma Metropolitana-Xochimilco, Mexico City, Mexico; St. Jude Children's Research Hospital, United States of America

## Abstract

Acute coronary syndromes (ACS) may be triggered by acute infections. Systemic production of interferon gamma (IFN-γ) is induced during infection and regulates the production of matrix metalloproteinases (MMPs) and their inhibitors (TIMPs), both important in plaque stability. This study evaluates the effect of IFN-γ on the MMPs/TIMP-1 ratio in cultured monocytes from 30 patients with stable coronary artery disease (CAD), 30 with unstable angina (UA) or non-ST-segment elevation myocardial infarction (NSTEMI), and 30 healthy blood donors. Supernatant concentrations of MMP-1, -2, -9, and TIMP-1 were measured by enzyme-linked immunoassays. Basal concentration of MMP-1 and TIMP-1 was similar between groups, while MMP-2 was higher in healthy individuals and MMP-9 in patients with UA/NSTEMI. Upon IFN-γ stimulation, MMP-9 secretion increased in all groups, while TIMP-1 decreased only in patients with CAD, which in turn result in a strikingly elevation in their mean MMP-9/TIMP-1 ratio. MMP-1/TIMP-1 and MMP-2/TIMP-1 ratios were <1.0 in basal conditions and after stimulation in all groups. Our results suggest that nonstimulated monocytes from patients with stable CAD show a similar behavior than those from healthy individuals. However, stimulation with IFN-γ induces an increase on the MMP-9/TIMP-1 ratio as high as that found in patients with ACS. Thus, it may bring biological plausibility to the association between acute infections and the development of ACS.

## Introduction

Atherosclerotic coronary artery disease (CAD) is the leading cause of death and a main source of morbidity worldwide [1,2]. Nowadays, it is clear that inflammation is important in CAD, in which circulating monocytes and tissue-invading macrophages play a role in the maintenance of plaque’s homeostasis [3]. Nonetheless, transition from plaque stability to instability is barely understood. In support to the existence of immune-based mechanisms, growing evidence suggests that acute coronary syndromes (ACS) could be triggered by infection [4]. The original interest in chronic bacterial infections as precipitants of myocardial infarction (MI) and stroke has been moving forward to acute respiratory infections with an emphasis on influenza viruses. Indeed, several epidemiological studies support a temporal association between acute respiratory virus infections and the development of ACS, after adjustment for potential environmental confounding factors [5–7].

Apart from the ecological evidence linking acute respiratory infections with ACS, mechanisms underlying this association are unclear. The currently favored mechanism points toward that acute infection may trigger plaque instability and rupture through a systemic response to inflammatory stimuli [8]. In this vein, infection by influenza induces the systemic production of inflammatory cytokines, especially interferon gamma (IFN-γ) which is a main regulator of the production of tissue matrix metalloproteinases (MMPs) and their endogenous inhibitors (TIMPs) by inflammatory cells such as circulating monocytes and infiltrating macrophages [9]. MMPs belong to a large family of zinc-dependent endopeptidases referred to numerically from 1 through 28; collectively, MMPs are capable of degrading all the extracellular matrix components of the fibrous cap that separates the necrotic core of the atherosclerotic lesion from blood flow in the arterial lumen [10]. Among this family of related proteases, MMP-1 (also called interstitial collagenase), MMP-2 (gelatinase-A), and MMP-9 (gelatinase-B) have been consistently described as significant contributors in several cardiovascular diseases including atherosclerosis, hypertension, CAD, and ACS [10]. In this regard, balance between synthesis and degradation of extracellular matrix components is crucial for the stability or vulnerability of atherosclerotic plaques [11]. Depending on the width, composition, and integrity of their fibrous cap, stable plaques may result in the development of stable CAD while vulnerable plaques may become disrupted, which in turn results in the development of ACS. Given their central role in tissue remodeling and inflammation, the effect of MMPs inhibition in the reduction of inflammation and the prevention of ACS is under study [10].

In patients with stable CAD, circulating leukocytes do not have increased expression of MMP-9 or TIMP-1 but an imbalance of the MMP-9/TIMP-1 ratio has been recently demonstrated in unstimulated monocytes from patients with ACS [12]. However, whether stimulation with IFN-γ actually induces an imbalance in the MMP/TIMP ratios in circulating monocytes from patients with stable CAD or ACS has not been elucidated.

The present study was aimed to evaluate the effect of IFN-γ on the secretion of MMP-1, MMP-2, MMP-9 and TIMP-1 as well as on the MMPs/TIMP-1 ratio, in cultured monocytes from patients with either stable CAD or ACS.

## Material and Methods

### Ethics statement

The study protocol was approved by the Research and Bioethics Commissions of the Instituto Nacional de Cardiología Ignacio Chávez. All participants provided a written informed consent, also approved by the Bioethics Commission. All procedures were conducted in accordance with the Declaration of Helsinki and local regulations.

### Study Population

This study was conducted in consecutive patients admitted to the Coronary Care Unit with diagnosis of unstable angina (UA) or non-ST-segment elevation MI (NSTEMI), in age- and gender-matched patients with an established diagnosis of stable CAD recruited from the Cardiology Outpatient Clinic, and in healthy blood donors.

Patients with a diagnosis of ACS were identified and classified based on clinical characteristics, electrocardiographic changes, and biochemical markers of cardiac necrosis (MB isoenzyme of creatine kinase or T-troponin) according to the definitions proposed by the American College of Cardiology [13]. Briefly, NSTEMI was defined as an ACS in which there is cardiac marker evidence of myocardial necrosis without new ST-segment elevation. UA was defined as angina pectoris (or equivalent type of ischemic discomfort) with any of the 3 following features: (a) angina occurring at rest and prolonged, usually greater than 20 minutes; (b) new-onset angina; (c) recent acceleration of angina reflected by an increase in its severity. Stable CAD was defined as ≥6 months history of chest/arm discomfort reproducible with physical exertion or stress, or >1 mm ST-segment depression on the exercise test electrocardiogram.

Patients with chronic inflammatory disease such as rheumatoid arthritis or systemic lupus erythematosus, recent MI or stroke (within the last 6 months), concurrent serious infection or trauma, atrial fibrillation, malignancy, end-stage renal or liver disease, or surgery (including catheterization) were not included.

### Laboratory assessments

For patients with UA/NSTEMI, blood samples were obtained at the time of admission to the Coronary Care Unit, while for those with stable CAD these were collected in their programmed medical consultation. In healthy individuals, samples were collected at the time of blood donation.

Five mL of peripheral blood were centrifuged (600 g for 15 min at 4° C), and sera were stored in aliquots at -75° C until use. Additionally, 5 mL of peripheral blood were centrifuged at 151 g during 30 min with Hystopaque-1077 (Sigma Aldrich, St Louis MO, USA), and the interface band of peripheral blood mononuclear cells (PBMC) was collected. Leukocytes were incubated in RPMI-1640 medium (GIBCO, Grand Island NY, USA) containing 10% heat-inactivated fetal calf serum (GIBCO), 25 mM HEPES buffer, L-glutamine, 100 IU/ml penicillin, 100 mg/ml streptomycin, and 1% nonessential amino acids. Cells were incubated for 24 hours at 37 °C in a humidified atmosphere with 5% of CO_2_. Non-adherent leukocytes were removed by washing with PBS, and adherent monocytes were recovered with cold PBS/EDTA and counted in a LH750 analyzer (Beckman Coulter, Brea CA, USA). 5 X 10^5^ monocytes/well were incubated in 24-well plates (Nunc, Houston TX, USA) with RPMI-1640 supplemented medium and stimulated with 10 ng/mL (corresponding to a concentration of 200 U/mL) of recombinant human IFN-γ (PeproTech, Rocky Hill NJ, USA) at 37° C. Previously, it has been shown that the minimum concentration of IFN-γ to induce significant decrements in the *in vitro* production of TIMP-1 is around 150 U/mL [14]. After 48 hours of incubation, supernatants were withdrawn and stored at −75° C until use.

Finally, serum levels of IFN-γ and supernatant concentrations of MMP-1, MMP-2, MMP-9 and TIMP-1 were measured by enzyme-linked immunosorbent assays using commercial kits (R&D Systems, Minneapolis MN, USA) according to instructions provided by the manufacturer.

### Statistical analyses

Frequencies and proportions were utilized to describe categorical data and differences were analyzed using the chi-square test. Continuous variables were expressed as median and interquartile range (IQR, 25^th^ to 75^th^ percentile) and compared using the Wilcoxon’s matched pairs test (basal *versus* stimulated) or the Mann-Whitney test (independent groups) as correspond. The Kruskal-Wallis with Dunn’s multiple comparisons tests were performed when more than 2 groups were compared.

The ratio of MMPs/TIMP-1 was first obtained for each patient and these individual data were used to obtain the arithmetic mean of the whole group and used to express the overall ratios. All analyses were two-sided, and significance was set at *p*<0.05. The GraphPad Prism ver. 4.02 (GraphPad Inc, San Diego CA, USA) statistical software was used for calculations.

## Results

The study was conducted in 30 patients with UA/NSTEMI (83% male, median age 61.5 years), 30 patients with stable CAD (83% male, median age 63 years), and 30 healthy blood donors. Demographic and clinical characteristics are described in Table 1. No differences were found in terms of hypertension and diabetes, previous use of aspirin and statins and other clinical data including lipid profile. However, the frequency of smoking (83 *versus* 53%; *p*<0.05) and the use of β-blockers (80 *versus* 47%; *p*<0.05) was higher in patients with UA/NSTEMI than in those with stable CAD.

Serum levels of IFN-γ were similar in patients with UA/NSTEMI or stable CAD (median 31.5, 23-39 *versus* 28, 15-35 pg/ml; *p* = ns), and these were higher to those found in blood donors (18, 10-31 pg/ml; *p*<0.05).

The concentrations of MMP and TIMP-1 in the supernatants of non-stimulated and IFN-γ-stimulated monocytes are described in Table 2. As noted, basal levels of MMP-1 were similar between groups, and this was not modified upon stimulation. Basal MMP-2 was higher in healthy controls than in both groups of patients, although its concentration significantly increased after IFN-γ stimulation only in the latter. In basal conditions, levels of MMP-9 were higher in patients with UA/NSTEMI than in the other groups; after stimulation, levels sharply increased in all groups. Levels of TIMP-1 were similar in the basal; after stimulation, TIMP-1 production was unchanged in cells from healthy donors but a three to five-fold drop was observed in monocytes from patients.

**Figure 1:**
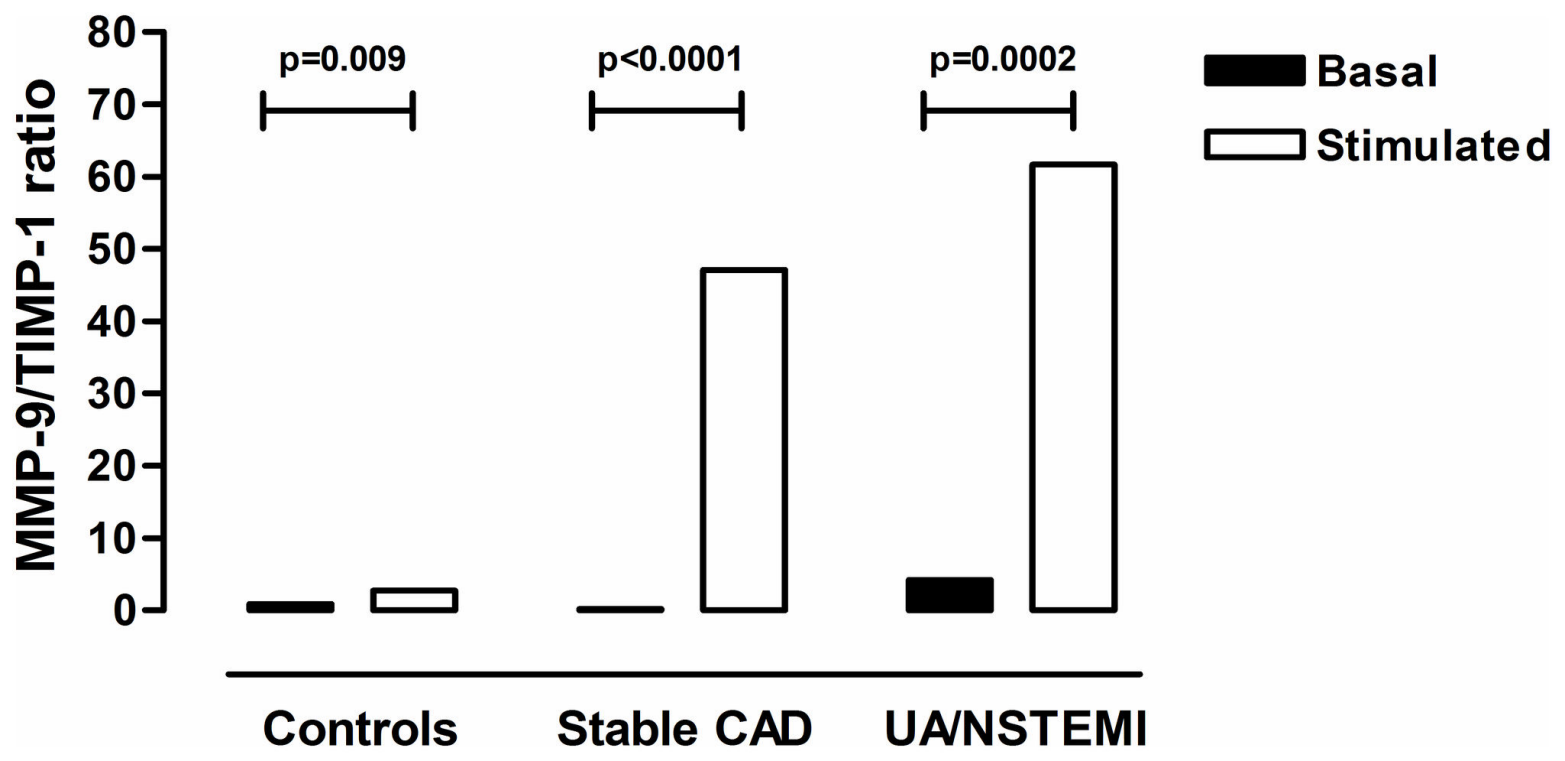
Matrix metalloproteinase-9 / Tissue inhibitor of matrix metalloproteinases 1 (MMP-9/TIMP-1) ratio in the supernatants of unstimulated (black column) and interferon gamma (IFN-γ) stimulated (white column) monocytes from healthy donors and patients with stable coronary artery disease (CAD) or unstable angina/non-ST-segment elevation myocardial infarction (UA/NSTEMI).

Regarding the MMP-1/TIMP-1 ratios, these were <1.0 in basal conditions and after stimulation in all groups. Similar results were found for the MMP-2/TIMP-1 ratio (data not shown). In contrast, mean MMP-9/TIMP-1 baseline ratio was imbalanced (>1.0) only in patients with UA/NSTEMI (ratio 4.4). As noted above, MMP-9 secretion increased in all groups while TIMP-1 decreased only in patients following the stimulation with IFN-γ. As a consequence, monocytes from both groups of patients strikingly increased their mean secreted MMP-9/TIMP-1 ratios (Figure 1). The MMP-9/TIMP-1 ratio from patients with UA/NSTEMI rose to 59 whilst this figure was 47 for patients with stable CAD but in healthy controls it only increased to 2.8.

**Table 1:** Characteristics of patients and healthy individuals.

		**UA/NSTEMI (n=30)**	**Stable CAD (n=30)**	***p***	**Healthy (n=30)**
Age, years		61.5 (39-80)	63 (38-81)	ns	54.8 (30-65)
Male, n (%)		25 (83)	25 (83)	ns	23 (77)
History of myocardial infarction, n (%)		16 (53)	19 (63)	ns	0
Cardiovascular risk factors, n (%)	Hypertension	21 (70)	21 (70)	ns	0
	Diabetes mellitus	15 (50)	17 (57)	ns	0
	Smoking	25 (83)	16 (53)	<0.05	17 (57)
Laboratory data	Cholesterol, mg/dL	159 (83-272)	164 (95-261)	ns	-
	LDL, mg/dL	99.8 (35-170)	93 (26-189)	ns	-
	HDL, mg/dL	36.3 (21-60)	37.1 (25-63)	ns	-
	Triglycerides, mg/dL	134 (59-535)	170.5 (64-386)	ns	-
	C-reactive protein, mg/L	3.3 (0.8-127)	2.1 (0.2-42)	<0.05	1 (0.2-7.5)
Medications at admission	Aspirin	29 (97)	28 (93)	ns	-
	Statins	27 (90)	28 (93)	ns	-
	β-blockers	24 (80)	14 (47)	<0.05	-

Data are expressed as median (range) unless otherwise specified.

UA, unstable angina; NSTEMI, non-ST segment elevation myocardial infarction; CAD, coronary artery disease.

**Table 2:** Concentration of matrix metalloproteinases (MMPs) and tissue inhibitor of matrix metalloproteinases 1 (TIMP-1) measured in the supernatants of non-stimulated (basal) and interferon-γ (IFN-γ) stimulated monocytes.

**Marker ng/mL**	**Condition**	**UA/NSTEMI**	**Stable CAD**	**Controls**	***p***
**MMP-1**	Basal	8.2 (5.8-16.5)	5.4 (1.5-10.8)	8 (3.5-14.2)	ns
	Stimulated	9.9 (5.3-13.1)	6.7 (4.6-9.9)	6.6 (4.2-11.3)	
**MMP-2**	Basal	1.5 (0-5.4)	2.8 (0.8-5)	5.8 (2.6-12.4)	0.002
	Stimulated	5.6 (0.9-11.8)**	8.4 (4.5-12.9)*	3.4 (1.3-11.7)	
**MMP-9**	Basal	187 (105-460)	30 (14-90)	120 (95-194)	0.0001
	Stimulated	1016 (649-1610)***	541 (193-1078)***	793 (427-1043)***	
**TIMP-1**	Basal	416 (139-951)	982 (568-1250)	594 (126-1395)	ns
	Stimulated	158 (7-465)*	181 (9-732)*	548 (277-1024)	

Data are expressed as the median and interquartile range (25^th^ to 75^th^ percentile).

UA, unstable angina; NSTEMI, non-ST segment elevation myocardial infarction; CAD, coronary artery disease.

The column on the far right denotes the “p value” when comparing basal concentrations throughout the three groups (Kruskal-Wallis tests). Meanwhile, significant differences between basal versus stimulated conditions for each group (Wilcoxon’s matched pairs tests) are represented with asterisks as follows: *p<0.05, **p<0.01, ***p>0.001.

## Discussion

In the present study, we have demonstrated that stimulation with IFN-γ, a prototypal antiviral and inflammatory cytokine, induces an imbalance on the MMP-9/TIMP-1 ratio in monocytes from patients with stable CAD as high as those from patients with ACS.

In the notion that stimulation with IFN-γ induces a dramatic imbalance on the MMP-9/TIMP-1 ratio, it is interesting that the serum levels of IFN-γ were significantly higher in patients with CAD than in healthy individuals. Although the present study did not evaluate the possible cellular sources of IFN-γ, it is largely known that this cytokine is synthesized by innate immune cells such as natural killer cells, monocytes, and macrophages as well as by several adaptive immune cells including CD4+ helper T cells [15]. Also, it is well-recognized that IFN-γ is highly expressed within atherosclerotic lesions thus locally increasing the recruitment of inflammatory cells, impairing reverse cholesterol transport, and enhancing the production of MMPs and other inflammatory cytokines [16]. Hence, a sudden increase in the production of circulating IFN-γ, perhaps as observed following an acute viral infection, could also play a role in destabilization of atherosclerotic plaque.

A seminal case series study including ^~^40,000 patients found that risk of adverse cardiovascular events is substantially higher after an episode of acute respiratory tract infection, with incidence ratios of 4.9 for MI and 3.19 for stroke [6]. Subsequently, the effect of influenza and influenza-like illnesses on triggering MI was assessed on a national cardiac disease registry database [7]. This study demonstrates that risk of MI is increased during days 1-3 after acute respiratory infection (incidence ratio 4.19), with the effect tapering over time. Moreover, influenza vaccine may reduce the risk to develop cardiovascular events in patients hospitalized due to ACS [17]. The PROBE study included 439 patients with an ACS who were randomly allocated to receive, or not, influenza vaccine; after 12 months of follow-up, a significant reduction (hazard ratio of 0.70) in the occurrence of major adverse cardiovascular events including death and hospitalization for ACS, stroke or heart failure was observed in those patients who received the influenza vaccine [17]. In a similar way, two randomized-controlled studies have addressed the effects of the influenza vaccine in patients with stable CAD [18,19]. The FLUVACS study found a significant reduction in cardiovascular mortality and other major adverse cardiovascular events following the use of influenza vaccine [18], whilst the FLUCAD study found no difference in cardiovascular deaths although the occurrence of coronary ischemic events was slightly lower in those receiving the influenza vaccine [19].

The relationship between influenza virus and the development of ACS is still unclear despite the aforementioned epidemiological observations [20]. The progression from stable CAD into an ACS is mainly determined by the integrity of the atherosclerotic plaque. In this regard, degradation of extracellular matrix within the atherosclerotic plaque is largely determined by the balance between the MMPs and their endogenous TIMPs. Increased levels and activity of MMPs are found in inflamed atherosclerotic plaques [21,22], although increased serum concentrations of MMPs and low levels of TIMPs are also found in patients with ACS [23]. Even when macrophage-derived foam cells within the plaque are a prominent source of MMP, Brunner et al. recently demonstrated an imbalance of MMP-9/TIMP-1 in the circulating monocytes isolated from patients with ACS [12]. They suggest that increased levels of serum MMPs in patients with ACS might result not only from the local liberation of ruptured plaques but also from circulating monocytes, thus reflecting a systemic inflammatory condition. These results are in line with other observations on the role of the increased MMPs activity and the MMPs/TIMP proteolytic imbalance in ventricular remodeling after an ACS [24–26].

The mechanisms underlying the observed differences in TIMP-1 production after stimulation in monocytes from healthy donors compared to patients with CAD either stable or unstable are not clear. However, this could be the result of an altered milieu of pro- and anti-inflammatory cytokines in individuals with clinically evident CAD [12,27].

Our results indicate that circulating monocytes from patients with chronic stable CAD are primed, and display a functional phenotype that could trigger plaque instability when they are stimulated with IFN-γ, a prototypal inflammatory cytokine massively released following an infection with influenza virus. We suggest that our results bring biological plausibility to the well-known association between acute infections with influenza virus and the development of ACS.

There are potential limitations to the present study. First, our results deserve to be studied in depth to appraise the clinical relevance of these findings. Second, longitudinal studies including a large number of patients may be necessary to better understand causality.

In conclusion, unstimulated monocytes from patients with ACS display an imbalance in the MMP-9/TIMP-1 ratio, whilst cells from patients with stable CAD show a similar behavior than those from healthy individuals. However, stimulation with IFN-γ induces a striking imbalance on the MMP-9/TIMP-1 ratio in the monocytes from patients with stable CAD as high as those from patients with ACS. These data may account for an explanation of the association between acute respiratory infections and the development of ACS in individuals with no evidence of a vulnerable plaque.

## References

[1] Hanson MA, Fareed MT, Argenio SL, Agunwamba AO, Hanson TR (2013) Coronary artery disease. Prim Care 40: 1-16. doi:10.1016/j.pop.2012.12.001. PubMed: 23402459. 23402459 10.1016/j.pop.2012.12.001

[2] Sánchez-Diaz CJ, García-Badillo E, Sánchez-Ramírez CJ, Juárez U, Martínez-Sánchez C (2012) Clinical characteristics, process of care and outcomes among Mexican, Hispanic and non-Hispanic white patients presenting with non-ST elevation acute coronary syndromes: Data from RENASICA and CRUSADE registries. Arch Cardiol Mex 82: 14-21. PubMed: 22452861. 22452861

[3] Zhou J, Chew M, Ravn HB, Falk E (1999) Plaque pathology and coronary thrombosis in the pathogenesis of acute coronary syndromes. Scand J Clin Lab Invest Suppl 230: 3-11. PubMed: 10389196. 10389196

[4] Siriwardena AN (2012) Increasing evidence that influenza is a trigger for cardiovascular disease. J Infect Dis 206: 1636-1638. doi:10.1093/infdis/jis598. PubMed: 23048169. 23048169 10.1093/infdis/jis598

[5] Warren-Gash C, Bhaskaran K, Hayward A, Leung GM, Lo SV et al. (2011) Circulating influenza virus, climatic factors, and acute myocardial infarction: a time series study in England and Wales and Hong Kong. J Infect Dis 203: 1710-1718. doi:10.1093/infdis/jir171. PubMed: 21606529. 21606529 10.1093/infdis/jir171 PMC3100509

[6] Smeeth L, Thomas SL, Hall AJ, Hubbard R, Farrington P et al. (2004) Risk of myocardial infarction and stroke after acute infection or vaccination. N Engl J Med 351: 2611-2618. doi:10.1056/NEJMoa041747. PubMed: 15602021. 15602021 10.1056/NEJMoa041747

[7] Warren-Gash C, Hayward AC, Hemingway H, Denaxas S, Thomas SL et al. (2012) Influenza infection and risk of acute myocardial infarction in England and Wales: a CALIBER self-controlled case series study. J Infect Dis 206: 1652-1659. doi:10.1093/infdis/jis597. PubMed: 23048170. 23048170 10.1093/infdis/jis597 PMC3488196

[8] Werba JP, Veglia F, Amato M, Baldassarre D, Massironi P et al. (2008) Patients with a history of stable or unstable coronary heart disease have different acute phase responses to an inflammatory stimulus. Atherosclerosis 196: 835-840. doi:10.1016/j.atherosclerosis.2007.01.033. PubMed: 17335831. 17335831 10.1016/j.atherosclerosis.2007.01.033

[9] Zhang L, Liao MF, Tian L, Zou SL, Lu QS et al. (2011) Overexpression of interleukin-1β and interferon-γ in type I thoracic aortic dissections and ascending thoracic aortic aneurysms: possible correlation with matrix metalloproteinase-9 expression and apoptosis of aortic media cells. Eur J Cardiothorac Surg 40: 17-22. doi:10.1016/j.ejcts.2010.09.019. PubMed: 21349736. 21349736 10.1016/j.ejcts.2010.09.019

[10] Bench TJ, Jeremias A, Brown DL (2011) Matrix metalloproteinase inhibition with tetracyclines for the treatment of coronary artery disease. Pharmacol Res 64: 561-566. doi:10.1016/j.phrs.2011.05.002. PubMed: 21624471. 21624471 10.1016/j.phrs.2011.05.002

[11] Halvorsen B, Otterdal K, Dahl TB, Skjelland M, Gullestad L et al. (2008) Atherosclerotic plaque stability--what determines the fate of a plaque? Prog Cardiovasc dis 51: 183-194. 19026853 10.1016/j.pcad.2008.09.001

[12] Brunner S, Kim JO, Methe H (2010) Relation of matrix metalloproteinase-9/tissue inhibitor of metalloproteinase-1 ratio in peripheral circulating CD14+ monocytes to progression of coronary artery disease. Am J Cardiol 105: 429-434. doi:10.1016/j.amjcard.2009.10.013. PubMed: 20152234. 20152234 10.1016/j.amjcard.2009.10.013

[13] Cannon CP, Battler A, Brindis RG, Cox JL, Ellis SG et al. (2001) American College of Cardiology key data elements and definitions for measuring the clinical management and outcomes of patients with acute coronary syndromes. A report of the American College of Cardiology Task Force on Clinical Data Standards (Acute Coronary Syndromes Writing Committee). J Am Coll Cardiol 38: 2114-2130. doi:10.1016/S0735-1097(01)01702-8. PubMed: 11738323. 11738323 10.1016/s0735-1097(01)01702-8

[14] Shapiro SD, Campbell EJ, Kobayashi DK, Welgus HG (1990) Immune modulation of metalloproteinase production in human macrophages. Selective pretranslational suppression of interstitial collagenase and stromelysin biosynthesis by interferon-gamma. J Clin Invest 86: 1204-1210. doi:10.1172/JCI114826. PubMed: 2170447. 2170447 10.1172/JCI114826 PMC296850

[15] Pedersen ER, Midttun Ø, Ueland PM, Schartum-Hansen H, Seifert R et al. (2011) Systemic markers of interferon-γ-mediated immune activation and long-term prognosis in patients with stable coronary artery disease. Arterioscler Thromb Vasc Biol 31: 698-704. doi:10.1161/ATVBAHA.110.219329. PubMed: 21183733. 21183733 10.1161/ATVBAHA.110.219329

[16] McLaren JE, Ramji DP (2009) Interferon gamma: a master regulator of atherosclerosis. Cytokine Growth Factor Rev 20: 125-135. doi:10.1016/j.cytogfr.2008.11.003. PubMed: 19041276. 19041276 10.1016/j.cytogfr.2008.11.003

[17] Phrommintikul A, Kuanprasert S, Wongcharoen W, Kanjanavanit R, Chaiwarith R et al. (2011) Influenza vaccination reduces cardiovascular events in patients with acute coronary syndrome. Eur Heart J 32: 1730-1735. doi:10.1093/eurheartj/ehr004. PubMed: 21289042. 21289042 10.1093/eurheartj/ehr004

[18] Gurfinkel EP, Leon de la Fuente R, Mendiz O, Mautner B (2004) Flu vaccination in acute coronary syndromes and planned percutaneous coronary interventions (FLUVACS) Study. Eur Heart J 25: 25-31. doi:10.1016/j.ehj.2003.10.018. PubMed: 14683739. 14683739 10.1016/j.ehj.2003.10.018

[19] Ciszewski A, Bilinska ZT, Brydak LB, Kepka C, Kruk M et al. (2008) Influenza vaccination in secondary prevention from coronary ischaemic events in coronary artery disease: FLUCAD study. Eur Heart J 29: 1350-1358. doi:10.1093/eurheartj/ehm581. PubMed: 18187561. 18187561 10.1093/eurheartj/ehm581

[20] Natarajan P, Cannon CP (2011) Myocardial infarction vaccine? Evidence supporting the influenza vaccine for secondary prevention. Eur Heart J 32: 1701-1703. doi:10.1093/eurheartj/ehr053. PubMed: 21406438. 21406438 10.1093/eurheartj/ehr053

[21] Choudhary S, Higgins CL, Chen IY, Reardon M, Lawrie G et al. (2006) Quantitation and localization of matrix metalloproteinases and their inhibitors in human carotid endarterectomy tissues. Arterioscler Thromb Vasc Biol 26: 2351-2358. doi:10.1161/01.ATV.0000239461.87113.0b. PubMed: 16888239. 16888239 10.1161/01.ATV.0000239461.87113.0b

[22] Sluijter JP, Pulskens WP, Schoneveld AH, Velema E, Strijder CF et al. (2006) Matrix metalloproteinase 2 is associated with stable and matrix metalloproteinases 8 and 9 with vulnerable carotid atherosclerotic lesions: a study in human endarterectomy specimen pointing to a role for different extracellular matrix metalloproteinase inducer glycosylation forms. Stroke 37: 235-239. PubMed: 16339461. 16339461 10.1161/01.STR.0000196986.50059.e0

[23] Cheng M, Hashmi S, Mao X, Zeng QT (2008) Relationships of adiponectin and matrix metalloproteinase-9 to tissue inhibitor of metalloproteinase-1 ratio with coronary plaque morphology in patients with acute coronary syndrome. Can J Cardiol 24: 385-390. doi:10.1016/S0828-282X(08)70602-0. PubMed: 18464944. 18464944 10.1016/s0828-282x(08)70602-0 PMC2643141

[24] Kelly D, Khan SQ, Thompson M, Cockerill G, Ng LL et al. (2008) Plasma tissue inhibitor of metalloproteinase-1 and matrix metalloproteinase-9: novel indicators of left ventricular remodelling and prognosis after acute myocardial infarction. Eur Heart J 29: 2116-2124. doi:10.1093/eurheartj/ehn315. PubMed: 18614523. 18614523 10.1093/eurheartj/ehn315 PMC2941717

[25] Kelly D, Cockerill G, Ng LL, Thompson M, Khan S et al. (2007) Plasma matrix metalloproteinase-9 and left ventricular remodelling after acute myocardial infarction in man: a prospective cohort study. Eur Heart J 28: 711-718. doi:10.1093/eurheartj/ehm003. PubMed: 17339265. 17339265 10.1093/eurheartj/ehm003 PMC2202923

[26] Ahmed SH, Clark LL, Pennington WR, Webb CS, Bonnema DD et al. (2006) Matrix metalloproteinases/tissue inhibitors of metalloproteinases: relationship between changes in proteolytic determinants of matrix composition and structural, functional, and clinical manifestations of hypertensive heart disease. Circulation 113: 2089-2096. doi:10.1161/CIRCULATIONAHA.105.573865. PubMed: 16636176. 16636176 10.1161/CIRCULATIONAHA.105.573865

[27] de Oliveira RT, Mamoni RL, Souza JR, Fernandes JL, Rios FJ et al. (2009) Differential expression of cytokines, chemokines and chemokines receptors in patients with coronary artery disease. Int J Cardiol 136: 17-26. doi:10.1016/j.ijcard.2008.04.009. PubMed: 18617279. 18617279 10.1016/j.ijcard.2008.04.009

